# Germline specific genes increase DNA double-strand break repair and radioresistance in lung adenocarcinoma cells

**DOI:** 10.1038/s41419-024-06433-y

**Published:** 2024-01-12

**Authors:** Wenqing Liu, Jan Willem Bruggeman, Qijing Lei, Ans M. M. van Pelt, Jan Koster, Geert Hamer

**Affiliations:** 1 Reproductive Biology Laboratory, Center for Reproductive Medicine, Amsterdam UMC, University of Amsterdam, Amsterdam, The Netherlands; 2 Amsterdam Reproduction and Development Research Institute, Amsterdam, The Netherlands; 3 Center for Experimental and Molecular Medicine, Laboratory of Experimental Oncology and Radiobiology, Amsterdam UMC, University of Amsterdam, Amsterdam, The Netherlands

**Keywords:** Oncogenes, Differentiation

## Abstract

In principle, germline cells possess the capability to transmit a nearly unaltered set of genetic material to infinite future generations, whereas somatic cells are limited by strict growth constraints necessary to assure an organism’s physical structure and eventual mortality. As the potential to replicate indefinitely is a key feature of cancer, we hypothesized that the activation of a “germline program” in somatic cells can contribute to oncogenesis. Our group recently described over one thousand germline specific genes that can be ectopically expressed in cancer, yet how germline specific processes contribute to the malignant properties of cancer is poorly understood. We here show that the expression of germ cell/cancer (GC) genes correlates with malignancy in lung adenocarcinoma (LUAD). We found that LUAD cells expressing more GC genes can repair DNA double strand breaks more rapidly, show higher rates of proliferation and are more resistant to ionizing radiation, compared to LUAD cells that express fewer GC genes. In particular, we identified the HORMA domain protein regulator TRIP13 to be predominantly responsible for this malignant phenotype, and that TRIP13 inhibition or expression levels affect the response to ionizing radiation and subsequent DNA repair. Our results demonstrate that GC genes are viable targets in oncology, as they induce increased radiation resistance and increased propagation in cancer cells. Because their expression is normally restricted to germline cells, we anticipate that GC gene directed therapeutic options will effectively target cancer, with limited side effects besides (temporary) infertility.

## Introduction

The germline is a unique lineage of cells that can be defined as all cells that have the possibility to propagate their genome to subsequent generations. This includes embryonic stem cells, primordial germ cells and germ stem cells, up to haploid mature gametes. Germline cells have properties that are often unique to their specific stage of germ cell development, such as stem cell proliferation and differentiation, meiosis, and gamete formation. Together these properties contribute to the capacity of the germline to pass a relatively intact genome to future generations. In contrast, somatic cells are restricted to only one generation and are not passed to future generations. Somatic cell death is ensured by senescence, which prevent uncontrollable cell growth [1]. In cancer, this safeguard is circumvented and escaping senescence is an intrinsic hallmark of cancer [2, 3]. When it fails, a cell acquires a defining trait of the germline: the ability not to age. As the pathways that allow for escaping senescence are embedded in the genome for germline-specific use, it would be much easier for a developing cancer cell to aberrantly activate dormant processes, rather than evolve new pathways entirely [4]. It is thus plausible that cancer cells utilize germline-specific processes to escape senescence. This is one of many examples of how a cancer cell may hijack germline specific processes to its benefit, more of which are discussed by [5].

We recently described a class of over one thousand germline-cancer genes (GC genes), which are defined as genes that are normally specific to the germline but are ectopically expressed in cancer [6, 7]. GC genes are involved in a wide variety of germline processes, including meiosis, gene regulation and DNA repair [6, 7]. In cancer, GC gene expression is observed in all of the 33 investigated tumor types of The Cancer Genome Atlas (TCGA) [8], but some cancers express more GC-genes than others. We previously found that the ectopic expression of GC genes in cancer is associated with a poor clinical prognosis in lung adenocarcinoma [7]. Similar associations have been found by others in non-small cell lung cancer, prostate cancer and renal clear cell carcinoma [9–14]. Despite these clinical observations, little is known about how germline specific processes may contribute to the malignant properties of cancer.

We here aim to investigate the relationship between GC gene expression and malignancy, as defined by therapy resistance, proficient DNA repair and proliferation rate. To this end, we used our list of GC-genes to create a model that allowed us to compare the phenotype of cancer cells that express many GC genes to cancer cells that express few GC genes. We tested the hypothesis that the observed clinical outcomes associated with more aggressiveness and resistance to therapy [9–14] can be observed in our model as well. In addition, we propose that one gene, TRIP13, is predominantly responsible for the malignant phenotype observed in some cell lines.

## Materials and methods

### Selection of cell lines as a model for comparing the effect of GC genes

**Fig. 1:**
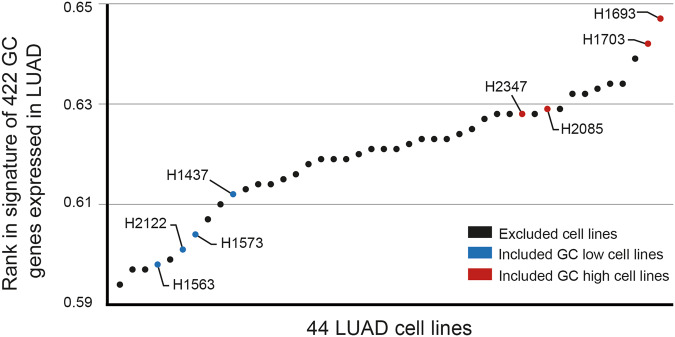
GC-signature scores of 44 lung adenocarcinoma (LUAD) cell lines. Every dot represents one cancer cell line. Red dots represent cell lines that we included in our analysis in order to compare the phenotype between high- versus low expression of GC-genes. H2085 and H1573 did not survive the standardized culture conditions and were not included for further analysis.

To investigate the effect of GC gene expression in cancer on response to chemotherapy and irradiation, induced double-stranded break repair and proliferation, we selected 4 lung adenocarcinoma (LUAD) cell lines with high GC gene expression (GC_high_) and 4 LUAD cell lines with low GC gene expression (GC_low_). The selection of groups was made by using R2 [15] to obtain LUAD-specific signature scores for each LUAD cell line in the Cancer Cell Line Encyclopaedia (CCLE) [16]. This score represents the average percentile of GC gene expression ranks for each cell line, and thus takes into account the high expression levels of GC genes and the low expression of non-GC genes. Using this approach, we previously reported that LUAD contains the largest variation of GC gene expression of all cancer cell lines in the CCLE, allowing for comparison of phenotypical traits within one type of cancer [7]. To specifically rank LUAD cell lines based on GC gene expression levels, we derived a new signature score based on 422 GC genes that are expressed in LUAD (Fig. 1) [6, 7]. In order to minimize baseline differences between cell lines, we applied several additional criteria. First, the cell lines must be epithelial and adherent. Second, they must be from the same source United States’ National Cancer Institute (NCI). Third, the cells must be cultured in identical growth medium (RPMI-1640). In order to adequately compare phenotypes, the top 4 and bottom 4 cell lines that fulfill these criteria were selected (Fig. 1 & Supplementary Data 1). These 8 LUAD cell lines were commercially obtained from the American Type Tissue Collection (ATCC), of which H2085 and H1573 did not survive standardized culture conditions, and were excluded from further analysis (Table 1).

**Table 1:** Included LUAD cell lines and characteristics.

GC category	Cell line	ATCC ID	Patient	FBS	Viability in cell culture	Protein-altering mutations in KRAS, NRAS, EGFR or TP53
Low	NCI-H1563	CRL-5875	Male, non-smoker, age unknown	10% FBS	Viable	–
	NCI-H2122	CRL-5985	46 y/o Caucasian female, 30 PY	10% FBS	Viable	KRAS: G12C TP53: C176F TP53: Q16L
	NCI-H1573	CRL-5877	35 y/o Caucasian female, 15 PY	5% FBS	Not viable	KRAS: G12A TP53: R248L
	NCI-H1437	CRL-5872	60 y/o Caucasian male, 70 PY	10% FBS	Viable	TP53: R267P
High	NCI-H2347	CRL-5942	54 y/o Caucasian female, non-smoker	10% FBS	Viable	KRAS: L19F NRAS: Q71R TP53: T125T
	NCI-H2085	CRL-5921	45 y/o male, smoking status unknown	10% FBS	Not viable	TP53: Y220C
	NCI-H1703	CRL-5889	54 y/o Caucasian male, 50 PY	10% FBS	Viable	–
	NCI-H1693	CRL-5887	55 y/o Caucasian female, 80 PY	5% FBS	Viable	–

*FBS* foetal bovine serum, *ATCC* American Type Culture Collection, *PY* pack years.

### Cell culture

Cells were maintained in 5% CO_2_ at 37°C in RPMI-1640 medium (Thermo Fisher Scientific), supplemented with 5% foetal bovine serum (Thermo Fisher Scientific) for H1693 and H1573 or 10% for the other 6 cell lines. Other supplements were 1% HEPES (Gibco), 1% Pen-Strep (Gibco), and 2.2% glucose (Gibco). Cells were refreshed every 3 days and passaged routinely for use until passage 20. Cells in culture tested negative for mycoplasma contamination during the entire study.

### Clonogenic assay

Clonogenic assays were performed as described previously [17]. For each cell line, an appropriate number of cells was plated in triplicates in 6-well plates (3000 cells for H1563, H1703 & H1437, 6000 cells for H2347 & H2122, and 14.000 cells for H1693). 4 h after plating, cells were exposed to 0 to 8 Gy of ionizing radiation (IR) in a CellRad system (Precision X-Ray) or 0 to 8 μM cisplatin. Cells were cultured in 3 ml medium, 2 ml of which was gently replaced after 7 days. Once the negative control condition (i.e. 0 Gy or 0 μM cisplatin respectively) showed formation of colonies of 50 cells, which was after approximately 14 days for all cell lines, medium was removed and cells were gently washed with phosphate-buffered saline (PBS), and fixed and stained with 6% glutaraldehyde +0.5% crystal violet in PBS. The numbers of colonies with >50 cells were electronically counted with ImageJ and manually confirmed.

Instead of using increasing dosages of treatment, we used 4 conditions to assess cells’ ability to form new colonies: no treatment, 1 Gy of IR, 10 μmol of the TRIP13 inhibitor DCZ0415 (HY-130603, Bio-Connect) [18] and 1 Gy + 10 μM DCZ0415.

### Immunofluorescent staining of γ-H2AX and RAD51

Cells were cultured on multi Nunc Lab-Tek II chambered slides (Thermo Fisher Scientific) for 24 h, after which they were exposed to 1 Gy of irradiation in a CellRad system (Precision X-Ray). Cells were fixated at various time points after irradiation (30 min–24 h) in 4% paraformaldehyde for 10 min and subsequently permeabilized in PBS with 0.1% triton-X for 15 min. Non-specific adhesion sites were blocked for 45 min in 0.25% Tween-20/PBS with 1% bovine serum albumin, followed by the addition of primary antibodies against γ-H2AX (1:10.000, 05-636, Millipore) or RAD51 (1:50, PA5-27195, Thermo Fisher Scientific), or isotype immunoglobulin G in the case of negative controls. After overnight incubation at 4 °C, the cells were washed and incubated with the corresponding host-specific secondary antibodies goat anti mouse Alexa Fluor 488 (1:1.000, A11029, Thermo Fisher Scientific) or goat anti Rabbit Alexa Fluor 532 (1:1000, A11009, Life Technologies), and counterstained with DAPI. The slides were mounted with Prolong Gold anti-fade (Thermo Fisher Scientific) and visualized using a Leica DM5000B microscope. γ-H2AX and RAD51 foci within the cell nucleus were counted manually in at least 100 cells per condition. This was repeated 3 times for statistical analysis.

### Western blotting

Cells were seeded in 6-well plates. Once 80% confluency was reached, media was discarded and cells were washed twice with phosphate-buffered saline. Cells were treated with 1 mL 0.25% trypsin and incubated for 5 min, after which the corresponding media was added to dilute the trypsin. The mixture was pipetted from the plates to tubes. Proteins were extracted from the cells using a RIPA buffer and the addition of protease- and phosphatase-inhibitors. Protein expression was quantified with the Qubit Protein Assay Kit (Thermo Fisher Scientific). Western blot analysis was performed using the LI-COR Odyssey imaging system (LI-COR Biosciences) as previously reported [19]. The primary antibodies were mouse anti-TRIP13 (1:1000, ab128171, Abcam), rabbit anti-GAPDH (1:400; FL-335, Santa Cruz Biotechnology), mouse anti-TUBULIN (1:1000; T9026, Sigma), rabbit anti-RAD51 (1:1000; PA5-27195, Thermo Fisher Scientific) mouse anti-KU70 (1:1000; ab2172-500, Abcam), rabbit anti-Ligase IV (1:1000； ab193353, Abcam). Band intensities were assessed using Image Studio Lite (Version 5.2).

### Proliferation assay

Cells were cultured in 96 well-plates. After 4 h, cells were exposed to IR in a CellRad system and/or 10 μmol TRIP13 inhibitor DCZ0415. When cells reached 50–60% confluency, the 5-ethynyl-2’-deoxyuridine (EdU) was added to the culture for 2 h. Quantification of EdU-positive cells was performed using the Cell Proliferation Kit (C10337, Thermo Fisher Scientific) as previously described [20] and represented by the mean ± SEM of 3 independent experiments. Images were analyzed using Leica Application Suite X and counting of nuclei and EdU stains was performed electronically in ImageJ.

### Quantitative-real time PCR (Q-PCR)

Total RNA was extracted from control and treated cells using the RNeasy Mini Kit (Qiagen) according to the manufacturer’s protocol. The RNA samples (*n* = 3 for all cell lines) were reversely transcribed using the SensiFAST cDNA Synthesis Kit (Bioline). The synthesized cDNA was then used for Q-PCR reactions, using the Roche LightCycler 480 platform in a 384-well plate format. The Q-PCR reaction was performed in a 10ul volume system including 2X LightCycler 480 SYBR Green I Master (Roche). *GAPDH*, *ACTB* and *TUBA1C* were used as reference genes. The data were analyzed using the delta Ct method. The primers for Q-PCR analysis are listed in Supplementary Table 1.

### Cell scratch (wound healing) assay

Cells were cultured in Incucyte® image lock 96 well-plates (Satorius BA-04855). When the cells reached around 90% confluency, the Incucyte 96-well wound marker tool (Sartorius 4563) was used following the protocol provided by the manufacturer. After a scratch was automatically set, the cells were gently washed to remove detached cells and medium was refreshed. Wound healing was assessed using the Incucyte Live Cell Analysis System and quantified using ImageJ (version 2.0).

### Generation of knockout cell line with CRISPR-Cas9

The plasmids containing CRISPR targeting *TRIP13* (sc-404006-NIC; Santa Cruz Biotechnology) or Control CRISPR/cas9 (sc-418922; Santa Cruz Biotechnology) was delivered to the cells using a Neon electroporator (Thermo Fisher Scientific), following the manufacturer’s guidance.), The program used for electroporation was voltage 1100, width 20 ms and pulse 2. Two days after electroporation, GFP+ cells were sorted by fluorescence-activated cell sorter (FACS, BD Biosciences). Single GFP positive cells were cultured for seven to eight weeks, and colonies were screened by Sanger sequencing and Western blotting using the anti-TRIP13 antibody (1:1000; ab128171, Abcam).

### Generation of TRIP13 overexpression cell line

H1563 cells were transfected (Neon electroporator (Thermo Fisher Scientific) with *TRIP13* CRISPR Activation Plasmid (sc-404006-ACT; Santa Cruz Biotechnology) or Control CRISPR plasmid (sc-437275; Santa Cruz Biotechnology). After 48 h, transfected cells were selected using 1ug/mL puromycin (Enzo Life Sciences, ALX-380-028). *TRIP13* overexpression was screened by Q-PCR and Western blot analysis.

### Cell viability assay

Cells were seeded in triplicate in 96-well plates. At around 70–80% confluency the cells were treated with 10 uM DCZ0415. Cell viability was measured using the Alamar Blue assay (BUF012B, BIO-RAD) according to the manufacturer’s protocol.

### Statistical analysis

The data obtained were analyzed in Prism Graph Pad and Microsoft Excel. Two-sided T-tests were performed with α = 0.05, unless stated otherwise. Variance was tested for using the F-test of equality of variances, and the T-test without assuming equal variance was used in case of F > 0.05. Results were corrected for multiple testing using the Bonferroni correction where appropriate.

## Results

### GC_high_ cell lines are more resistant to IR

**Fig. 2:**
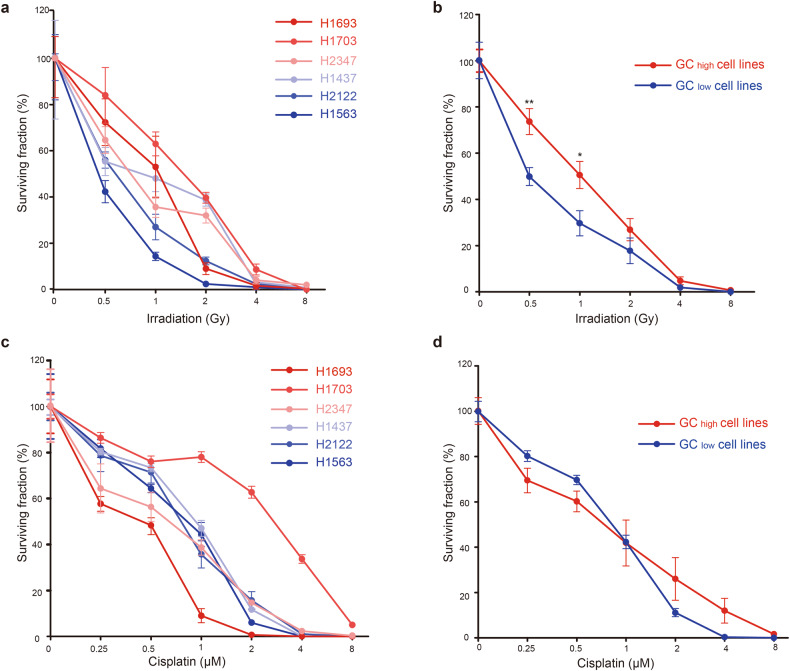
GC_high_ cell lines are more radioresistant than GC_low_ cell lines. **a** Survival curves of 6 LUAD cell lines in response to irradiation. **b** Mean survival curves of LUAD cell lines in response to irradiation grouped by GC_high_ cell lines (*n* = 3) and GC_low_ cell lines (*n* = 3). **c** Survival curves of 6 LUAD cell lines in response to cisplatin. **d** Mean survival curves of LUAD cell lines in response to cisplatin grouped by GC_high_ cell lines (*n* = 3) and GC_low_ cell lines (*n* = 3). *p < 0.05. **p < 0.01.

To investigate the association between GC gene expression and therapy resistance, six lung adenocarcinoma (LUAD) cell lines were grown in standardized culture conditions to be used as a model to test the effect of GC gene expression: three with high GC gene expression (GC_high_) and three with low GC gene expression (GC_low_) (Materials & methods). To investigate the association between GC gene expression and therapy resistance, we subjected all six cell lines to a clonogenic assay. This experiment results in surviving fraction of cells that is able to form new colonies after exposure to ionizing radiation (IR) or cisplatin, resulting in a survival curve of each cell line (Fig. 2). We observed that GC_high_ cell lines were more capable of colony formation following IR, compared to GC_low_ cell lines, especially at 0.5 Gy (*p* = 0.006) and 1 Gy (*p* = 0.02). While on average the GC_low_ cell lines are more sensitive to low dose irradiation than the GC_high_ cell lines, one GC_low_ cell line (H1437) appeared particularly resistant to IR above 1 Gy (Fig. 2a). For cisplatin, we did not observe a correlation between the cell lines’ ability to form new colonies and GC gene score (Fig. 2c/d).

### GC_high_ cell lines efficiently repair IR-induced DSBs

**Fig. 3:**
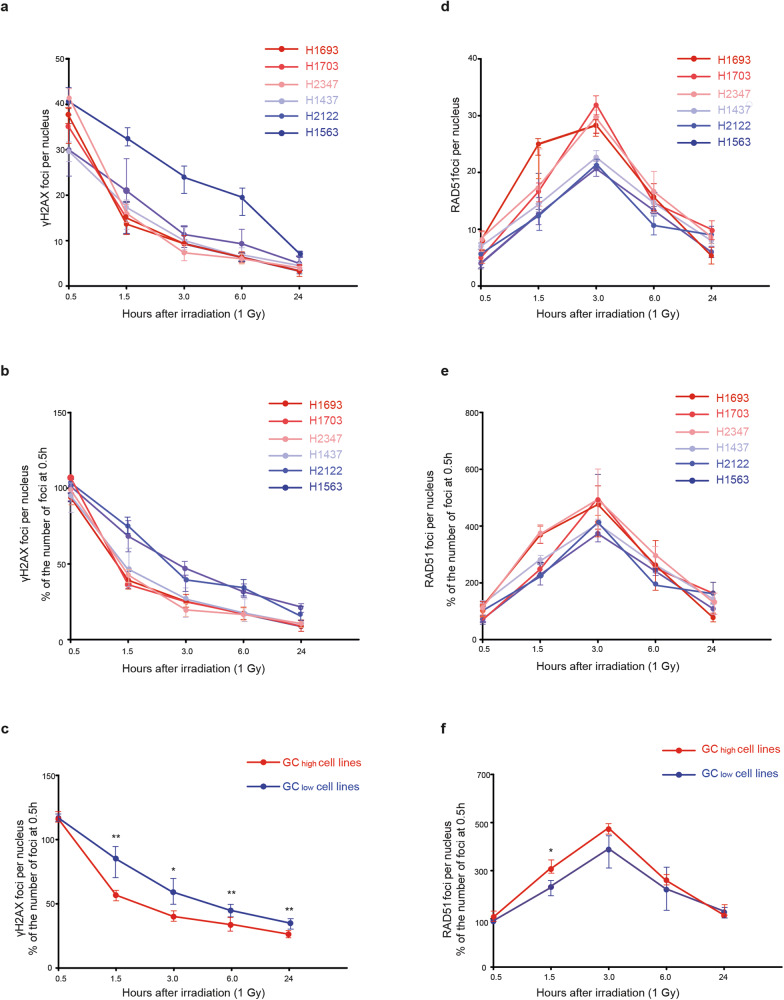
GC_high_ cell lines repair double-stranded breaks more efficiently than GC_low_ cell lines. **a** γ-H2AX foci after 1 Gy of irradiation at several time points in 6 LUAD cell lines. **b** γ-H2AX foci after 1 Gy of irradiation in 6 LUAD cell lines, relative to 30 min after exposure, averaged by GC gene category. **c** γ-H2AX foci after 1 Gy of irradiation in GC_high_ (red) and GC_low_ cell lines (blue), relative to 30 min after exposure. **d** RAD51 foci after 1 Gy of irradiation at several time points in 6 LUAD cell lines. **e** RAD51 foci after 1 Gy of irradiation in 6 LUAD cell lines, relative to 30 min after exposure, averaged by GC gene category. **f** RAD51 foci after 1 Gy of irradiation in GC_high_ (red) and GC_low_ cell lines (blue), relative to 30 min after exposure. *p < 0.05. **p < 0.01.

To investigate whether the increased resistance to IR observed in the GC_high_ cell lines is due to more efficient repair of DNA double-strand breaks (DSBs), we quantified the number of double-stranded breaks (DSBs) in all cell lines through γ-H2AX staining at 0.5, 1.5, 3, 6 and 24 h after exposure to 1 Gy of IR (Fig. 3a). We observed that the foci were resolved more slowly in two GC_low_ cell lines (H1563 and H2122), compared to the GC_high_ cell lines (Fig. 3b). The GC_low_ cell line H1437 clearly followed the DSB repair rate of the GC_high_ cell lines (Fig. 3b). Nevertheless, taken together, GC_high_ cell lines repair DNA significantly faster than GC_low_ cell lines (Fig. 3c).

### GC_high_ cell lines display more IR-induced RAD51 foci

In addition to γH2AX, to investigate differences in DSB repair via homologous recombination (HR), we quantified RAD51 foci formation and resolution at 0.5, 1.5, 3, 6 and 24 h after exposure to 1 Gy of IR. The number of RAD51 foci was lower in the three GC_low_ cell lines compared to the GC_high_ cell lines (Fig. 3d, e, Supplementary Fig. S1). Interestingly, in contrast to γH2AX, the GC_low_ but TRIP13 high cell line (H1437) did not follow RAD51 foci resolution of the GC_high_ cell lines. Taken together, GC_high_ cell lines repair DNA significantly faster than GC_low_ cell lines (Fig. 3c) and display more RAD51 foci (Fig. 3f).

To investigate whether the GC_high_ or GC_low_ cells may differentially repair DSBs via non-homologous end-joining (NHEJ), we treated the six cell lines with the previously characterized DNA-PKcs inhibitor NU7026 [21]. We measured its effect on cell survival after 1 Gy of irradiation and found that NU7026 reduced the cancer cell surviving fraction in all cell lines. Importantly, survival of the cell lines with a high GC gene score was not affected significantly different than survival of the cell lines with a low GC gene score (Supplementary Fig. S2A).

### GC_high_ cell lines maintain a higher rate of proliferation following IR

**Fig. 4:**
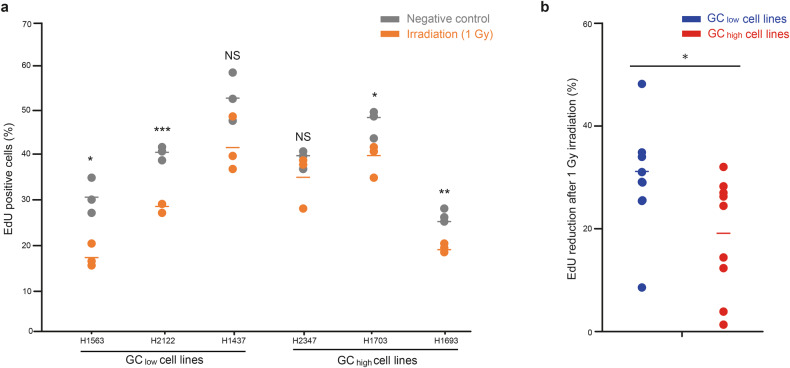
GC_high_ cell lines maintain a higher rate of proliferation following irradiation. **a** Percentage of 5-ethynyl-2’-deoxyuridine (EdU) positive cells of each LUAD cell line before and after 1 Gy of irradiation, shown in triplicate. **b** After irradiation (1 Gy), GC_high_ cell lines show a larger reduction in EdU absorption, compared to GC_low_ cell lines. NS not significant, *p < 0.05. **p < 0.01. ***p < 0.001.

To test for a difference in cell proliferation between GC_high_ and GC_low_ cell lines, we stained cells with DNA synthesis marker 5-ethynyl-2’-deoxyuridine (EdU). This assay showed that GC_high_ and GC_low_ cell lines did not differ in EdU incorporation (*p* = 0.43) (Fig. 4a). However, after exposure to 1 Gy of IR, GC_low_ cell lines absorbed 32% less EdU than their non-irradiated counterpart cells, while GC_high_ cell lines absorb only 19% less (*p* = 0.033, Fig. 4a/b). These results indicate that GC_high_ cell lines maintain a higher rate of proliferation following IR.

### GC_high_ cell lines show higher expression of pluripotency markers and higher invasion potential

To investigate the effect of GC genes on pluripotency we analyzed expression of *OCT4*, *Nanog* and *SOX2* using Q-PCR analysis of all our 6 LUAD cell lines (*n* = 3 for all cell lines). The GC_high_ cell lines showed a clear and significant increase in expression of these genes (Supplementary Fig. S3A–C). To quantify cell invasion potential, we performed a scratch assay using the Incucyte® imagelock 96-well plates and live cell imaging system (*n* = 3 for all cell lines). The GC_high_ cells appeared to display a higher invasion potential (Supplementary Fig. S3D, E).

### TRIP13 expression may be responsible for radioresistance

**Fig. 5:**
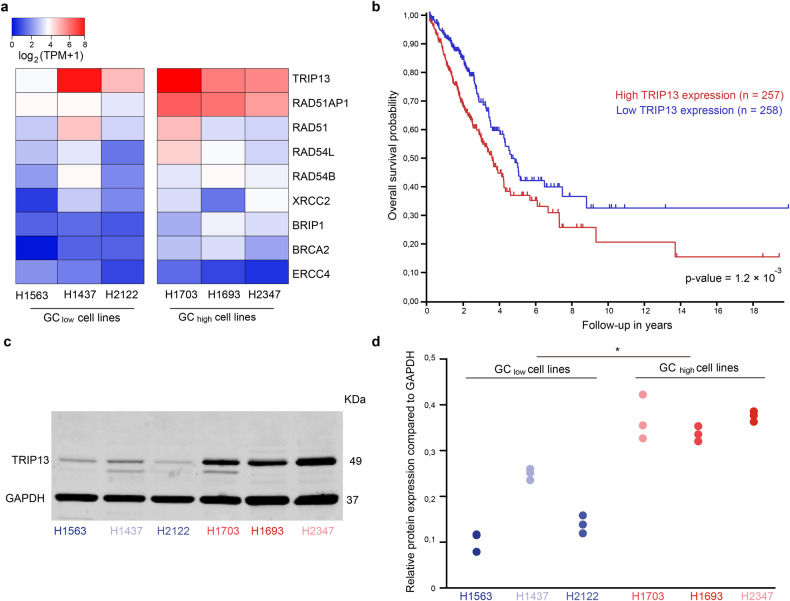
TRIP13 is most differentially expressed between GC_high_ and GC_low_ cell lines. **a** Heat map based on the RNA expression (transcripts per million) of 9 genes involved in meiosis and DSB repair in 6 LUAD cell lines. **b** High TRIP13 RNA expression in 515 LUAD patient samples correlates with poor prognosis (median, *p* = 0.001). **c** Western blot gel showing TRIP13 and GAPDH protein expression. **d** Protein quantification expressed as relative to GAPDH in 6 LUAD cell lines. *p < 0.05.

Because GC_high_ cell lines appear to be more radioresistant than GC_low_ cell lines, and more efficiently repair IR-induced DSBs, we hypothesized that this effect is induced by genes that are normally functional in meiotic recombination. Nine meiotic genes are associated with both gene ontology terms ‘meiosis’ (GO:0051321) and ‘double-strand break repair’ (GO:0006302), and their expression differs between the 6 LUAD cell lines (Fig. 5a). Of these, the gene TRIP13 was of particular interest because it (i) showed the highest expression in all cell lines, (ii) it showed the largest difference in expression between GC_low_ and GC_high_ cell lines, (iii) it could be confirmed as GC gene on the protein level using the Human Protein Atlas (http://www.proteinatlas.org), and (iv) is highly expressed in the GC_low_ cell line that behaves as GC_high_ (H1437) with regard to its relatively high resistance to irradiation and rapid DSB repair. Moreover, a retrospective survival analysis of 515 LUAD patients included in the Cancer Genome Atlas [8] shows that the RNA expression level of TRIP13 is associated with a poor prognosis (*p* = 0.001, Fig. 5b). We confirmed the differential expression of TRIP13 between GC_high_ and GC_low_ cell lines at the protein level using Western Blot analysis, quantified by relative expression compared to GAPDH (Fig. 5c/d). Among the GC_low_ cell lines, H1437 stood out again, as also observed in prior experiments.

### TRIP13 inhibition strongly impairs cell survival and proliferation

**Fig. 6:**
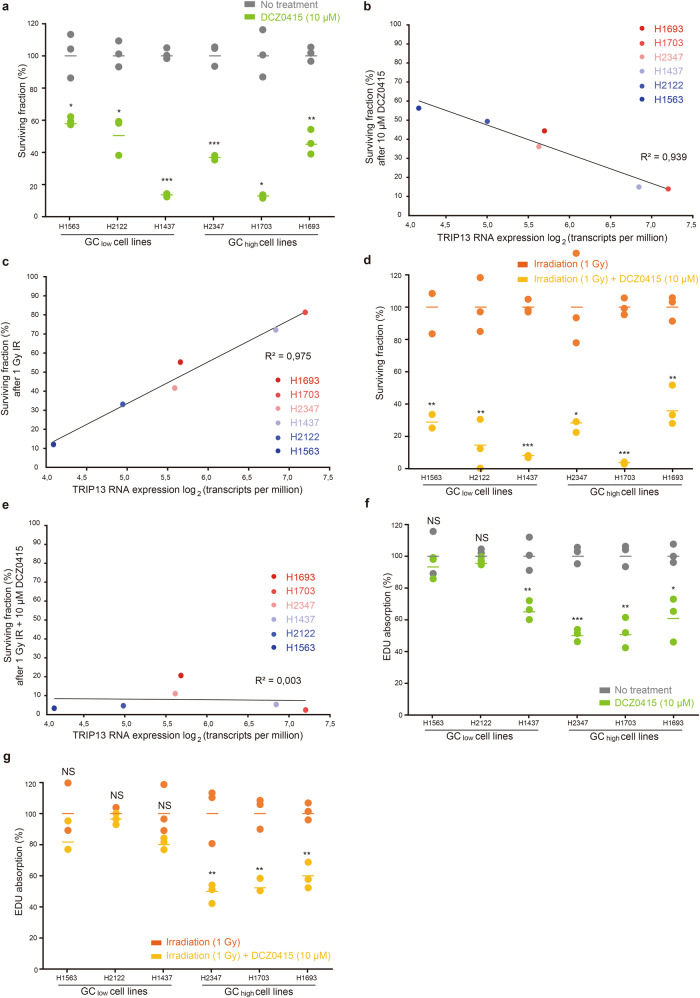
Inhibiting TRIP13 strongly impairs colony formation and proliferation. **a** Inhibiting TRIP13 with 10 µM DCZ0415 significantly reduces the surviving fraction in all cell lines (64%, CI_95%_ 55–73%), where 100% is defined as the average of three replicates not exposed to any treatment. **b** TRIP13 expression (Log_2_ (transcripts per million)) is strongly correlated with a reduced surviving fraction after treatment with 10 µM DCZ0415 (R^2^ = 0.94, *p* = 0.001). **c** TRIP13 expression is strongly correlated with an improved surviving fraction following 1 Gy of IR (R^2^ = 0.98, *p* < 0.001). **d** The addition of 10 µM DCZ0415 and 1 Gy of IR significantly decreased surviving fraction in all cell lines (81%, CI_95%_ 77–84%), where 100% is defined as the average of three replicates exposed to 1 Gy of IR alone. **e** Treatment with 1 Gy irradiation and 10 µM DCZ0415 strongly impairs the surviving fraction of all LUAD cell lines, independent of TRIP13 expression (R^2^ = 0.003, *p* = 0.92). **f** EdU absorption after treatment with 10 µM DCZ0415 was significantly reduced in all GC_high_ cell lines and in H1437, where 100% is defined as the average of three replicates not exposed to any treatment. **g** EdU absorption after treatment with 10 µM DCZ0415 and 1 Gy irradiation was significantly reduced in all GC_high_ cell lines, where 100% is defined as the average of three replicates exposed to 1 Gy of IR alone. NS not significant, *p < 0.05. **p < 0.01. ***p < 0.001.

To test whether the differences in proliferation and response to irradiation are affected by the expression of TRIP13, we repeated the aforementioned experiments with addition of DCZ0415, a newly discovered compound that has been shown to inhibit TRIP13 [18]. We measured the effect of inhibiting TRIP13 on cell survival and found that the addition of 10 µM DCZ0415 to the growth media reduced the cancer cell surviving fraction in all cell lines by an average of 64% (CI_95%_ 55–73%, Fig. 6a). Of all cell lines, the surviving fraction after TRIP13 inhibition was most decreased in H1703 and H1437, both of which show the highest TRIP13 expression of all 6 cell lines. To see whether the effect of DCZ0415 correlates with TRIP13 expression, we compared the reduction of the surviving fraction after treatment with DCZ0415 to the initial level of TRIP13 RNA expression. The reduction of the surviving fraction after treatment with 10 µM DCZ0415 indeed strongly correlates with the initial level of *TRIP13* RNA expression (Fig. 6b, R^2^ = 0.94).

As TRIP13 is involved in meiotic DSB repair [22], we proceeded to test the influence of DCZ0415 on the radioresistance of the six LUAD cell lines. TRIP13 expression is strongly correlated with the surviving fraction following 1 Gy of IR (Fig. 6c). Combining 1 Gy of IR treatment with 10 µM DCZ0415 was able to impair the surviving fraction by an average of 81% across all cell lines (CI_95%_ 77–84%), compared to 1 Gy of IR alone (Fig. 6d). This increase in impaired colony formation did not correlate with the initial *TRIP13* RNA expression (R^2^ = 0.003, Fig. 6e). In summary, TRIP13 inhibition by DCZ0415 correlates with a reduction of the surviving fraction, both with and without IR. In summary, TRIP13 inhibition by DCZ0415 correlates with a reduction of the surviving fraction, both with and without IR. TRIP13 expression appeared associated with increased radioresistance, and its inhibition by DCZ0415 led to a relatively stronger reduction of the surviving fraction of cells that express higher levels TRIP13.

Because it has been demonstrated that phosphorylation of TRIP13 at Y56 sensitizes head and neck cancer to the EGF receptor inhibitor cetuximab [23], we performed the clonogenic assay in all six cell lines, treated with or without cetuximab. We found that cetuximab indeed clearly affected survival of the high TRIP13 expressing cell lines (Supplementary Fig. S2B). However, although H2122 seemed unaffected by cetuximab, also the cell line H1563 (GC_low_, low TRIP13) was strongly affected by cetuximab. Hence, although cetuximab affects LUAD cells, the relation between this effect and GC gene or TRIP13 expression remains inconclusive.

To control that DCZ0415 specifically inhibits TRIP13, we used CRIPSR-CAS9 to remove *TRIP13* from one cell line. For this we chose the cell line H1703 (GC_high_, high TRIP13). Using single cell cloning and Sanger sequencing, we were able to pick up one clone that displayed two disrupted *TRIP13* alleles (Supplementary Fig. S4A–C). Using Western blot analysis, we found that the TRIP13 protein was removed in these cells (Supplementary Fig. S4D, E).

Using this cell line (TRIP13^KO^), we performed the cell viability assay to detect whether *TRIP13* removal would decrease sensitivity to DCZ0415. As control we used cells treated with scrambled guide RNAs during the CRISPR-CAS9 procedure. Removal of TRIP13 clearly decreased the response to DCZ0415, suggesting that DCZ0415 indeed specifically inhibits TRIP13 (Supplementary Fig. S5A).

In addition, we used the GC_low_ /low TRIP13 cell line H1563, to overexpress *TRIP13*. For this we used the TRIP13 CRISPR activation plasmid, including the synergistic activation mediator (SAM) transcription activation system, to overexpress TRIP13 or the CRISPR Activation plasmid encoding the deactivated Cas9 (dCas9) nuclease as control (Supplementary Fig. S4F–H). Using these cell lines, we performed the clonogenic assay to quantify the cells’ survival 14 days after irradiation with 2 Gy or without irradiation. (Supplementary Fig. S5B, C). Overexpression of TRIP13 indeed led to increased survival in response to IR.

### TRIP13 inhibition decreases RAD51 induction after irradiation

To investigate the mechanism by which TRIP13 is making the GC_high_ cell lines more resistant to IR, we performed Western blot analyses on all six cell lines after irradiation, with and without DCZ0415 treatment. First, we analyzed expression of HR marker RAD51. Except for H1563, irradiation induced RAD51 in all cells, irrespective of GC gene score. TRIP13 inhibition by DCZ0415 decreased the levels of RAD51 in all cells, quantified by relative expression compared to TUBULIN (Supplementary Fig. S6A, B). We performed the same experiment to investigate the presence of non-homologous end-joining (NHEJ) proteins KU70 and Ligase IV. In contrast to RAD51, DCZ0415 treatment did not affect expression of KU70 or Ligase IV, quantified by relative expression compared to GAPDH (Fig. S6C–E).

### TRIP13 correlates with cell proliferation before and after irradiation

Finally, in addition to measuring the colony formation ability of cancer cells, we also repeated the EdU proliferation assay after addition of DCZ0415 (Fig. 6f). This assay showed that addition of DCZ0415 led to a 15% reduction of EdU positive cells in GC_low_ cell lines, compared to 46% in GC_high_ cell lines (*p* < 0.001). H1437, which is a GC_low_ cell line with a high TRIP13 expression level, showed a 35% reduction in EdU absorption following the DCZ0415 treatment. Similarly, GC_high_ cell line H1703 shows a 49% reduction in EdU absorption following DCZ0415 treatment. We then repeated the EdU assay to observe the added effect of TRIP13 inhibition to irradiation. Compared to 1 Gy of IR alone, the addition of 10 µM DCZ0415 shows a significant decrease in EdU absorption in all GC_high_ cell lines, but not in GC_low_ cell lines (Fig. 6g). In conclusion, TRIP13 expression is associated with cell proliferation, and its inhibition by DCZ0415 led to a reduction in cell proliferation of cells that express higher levels TRIP13.

## Discussion

We here find that LUAD cells that express a relatively high number of GC genes (GC_high_) can rapidly repair DSBs, show higher rates of proliferation and are more resistant to IR, compared to LUAD cells that express a relatively low number of GC genes (GC_low_). Due to increased radioresistance of GC_high_ cell lines in response to irradiation, but not cisplatin, we speculate that this increased malignancy may be attributed to (pseudo-)meiotic activity that is encoded by GC genes. The DNA damage response in meiosis involves GC genes that, when upregulated by cancer cells, enhance their ability to withstand DSBs that are induced by IR. One gene strongly associated with increased IR resistance in our cells appeared to be TRIP13, and we here show that the putative TRIP13 inhibitor DCZ0415 leads to a decreased DNA damage response, which may allow for improved cancer treatment options.

In addition, we find that GC_high_ cells have higher expression of the pluripotency markers *OCT4*, *Nanog* and *SOX2*. Moreover, they show a higher invasion potential measured using the wound healing assay. However, since two cell lines, representing both the GC_high_ and GC_low_ groups, display a very high invasion potential, future research on both stemness and invasion potential is warranted.

Maybe the most well-known germline-specific feature is meiosis, the highly specialized cell division that ultimately results in the formation of haploid gametes [24]. Meiosis includes the induction of hundreds of DSBs that are required for crossover formation [25]. These DSBs are effectively dealt with by meiotic recombination, which not only requires genes involved in somatic homologous recombination, but also involves many meiosis specific genes. Among these meiosis specific genes are GC genes we previously identified, such as RAD51AP1, HORMAD1, DMC1 and SMC1β [6, 7]. While meiosis is tightly controlled in germ cells, the ectopic expression of such GC genes in cancer may result in the aberrant activation of pseudomeiotic processes, such as partial meiotic recombination or maybe even faulty assembly of the synaptonemal complex [26, 27]. For example, TEX12 is a gene that normally mediates synaptonemal complex assembly, but ectopic expression in somatic cells contributes to oncogenic centrosome amplifications [28]. Another example is Aurora kinase C (AURKC), which is required for the spindle assembly checkpoint during meiosis, but leads to increased migration and oncogenic transformation when ectopically expressed in somatic cells [29]. Ectopic expression of meiotic genes that are normally involved in cell cycle checkpoints or DNA damage repair could thus have a major influence on how cancer cells respond to DNA damaging agents, such as irradiation.

We observed that GC_high_ cell lines are more susceptible to DNA damage caused by IR, which predominately induces DSBs. Using immunofluorescence with γ-H2AX following irradiation, we found that DSB repair is indeed more efficient in GC_high_ cell lines. It could thus be hypothesized that GC genes induce partial meiotic recombinational repair of DSBs. From a bioinformatic analysis it indeed appeared that partial activation of meiotic recombinational processes can lead to increased repair of DSBs in cancer cells, which would ultimately lead to more genomic instability and further oncogenesis [26]. One gene involved in the DNA damage response that shows high differential expression between GC_high_ and GC_low_ cell lines is RAD51-associated protein 1 (RAD51AP1), which directs RAD51 towards DNA damage to initiate meiotic recombination [30]. Loss of RAD51AP1 leads to defective homologous recombination and genome instability [31], while upregulation of RAD51AP1 has been associated with a poor prognosis in several kinds of adenocarcinoma [32]. Indeed, we find that GC_high_ cell lines display higher amounts of RAD51 foci in response to irradiation induced DSBs. Because inhibition of non-homologous end-joining (NHEJ) does not significantly affect the GC_high_ cells more than the GC_low_ cells, it seems that expression of GC genes predominately has an effect on homologous recombination (HR).

Another meiotic and DNA repair response associated gene that shows a high differential expression between GC_high_ and GC_low_ cell lines is TRIP13 (Thyroid Hormone Receptor Interacting Protein 13), which is an AAA-ATPase that acts as a chaperone in a variety of cellular processes [33]. It is highly expressed during embryogenesis, in testicular tissue and a variety of cancers [22, 34]. While TRIP13 gene expression was not detectible in the GTEx data that was used to identify GC genes [6, 7], TRIP13 is expressed at a low yet functional level in mitosis, where its role is likely to disassemble the mitotic spindle checkpoint complex [35, 36]. The effects of TRIP13 inhibition are widespread because TRIP13 is a key orchestrator of the HORMA domain protein family, which includes the genes MAD2L2, MAD2L1, HORMAD1, HORMAD2, ATG13, ATG101, CMT2. These proteins regulate a variety of signaling processes, including mitosis, meiotic recombination, DNA damage repair, and autophagy [37]. HORMA domain proteins can be active or inactive, depending on the availability of their C-terminal binding site. When bound to a closure motif, the HORMA domain protein becomes active and able to bind a substrate. Reversion from the active to the inactive state occurs through TRIP13, together with MAD2 (or p31 ^comet^) [38]. One HORMA domain protein is MAD2L2 (or Rev7), a crucial component of the Shieldin complex that binds at DSBs sites to suppress recombination and allows for NHEJ to take place. The presence of TRIP13 during DSB repair leads to the inactivation of Rev7 and the disassembly of Shieldin complexes [39]. As a result, NHEJ is inhibited and recombination becomes the preferred DNA repair pathway [38–40]. Sustained expression of TRIP13, as is frequently observed in cancer, thus provides cancer cells with an alternative DNA repair mechanism through recombination [38]. During meiosis, recombination is promoted by TRIP13, as it facilitates depletion of the meiosis-specific HORMA domain proteins HORMAD1 and HORMAD2 from the chromosome axes as synapsis occurs, leading to further progression of meiotic recombination and cross-over formation [41, 42].

To understand the role of TRIP13 in meiosis, Li & Schimenti reduced TRIP13 expression in mice, leading to meiotic arrest due to accumulation of DNA damage in spermatocytes caused by dysfunctional recombination at the pachytene stage [22]. Later, Roig et al. were able to induce a more severe TRIP13 mutation that, in addition to impaired recombination, also leads to impaired synapsis of the homologous chromosomes and the lack of XY-body formation [43]. Despite the role of TRIP13 in mitosis, TRIP13-mutant mice were viable and looked no different from TRIP13-wildtype mice, except for reduced testis size. Humans with biallelic mutations in TRIP13 are highly susceptible to Wilms tumor and chromosome missegregation due to impairment of the spindle assembly checkpoint [44]. In addition, human TRIP13-mutant oocytes cannot complete meiosis [45]. On the other hand, upregulating TRIP13 in non-malignant cells leads to an oncogenic phenotype [21], which may be accounted for by two distinct mechanisms. First, sustained TRIP13 expression may lead to the inappropriate silencing of mitotic checkpoints, thereby allowing for rapid proliferation. Second, overexpression of TRIP13 may induce the function it has in meiotic prophase, inhibiting NHEJ and promoting of homologous recombination. As such, TRIP13 overexpression leads to the activation of HORMA domain proteins to induce chromosomal instability and is associated with a poor prognosis in several types of cancer [21, 46–48]. Based on these studies, as well as our observation that TRIP13 inhibition combined with irradiation largely reduces in vitro survival, we suggest that the role of overexpressed TRIP13 in cancer may result in the recombination phenotype, similar to its physiological role during the prophase of meiosis I. As TRIP13 expression is not strictly germ cell (or cancer) specific, this recombination-promoting phenotype would thus be a germ cell/cancer specific manifestation of TRIP13 overexpression.

In our experiments, TRIP13 is generally highly expressed in GC_high_ cell lines and lowly expressed in in GC_low_ cell lines. However, H1437 is a GC_low_ cell line that has a much higher TRIP13 expression than the other GC_low_ cell lines. From the clonogenic assay it indeed appears that H1437 is more radioresistant than the other 2 GC_low_ cell lines, and its survival curve follows the curve of GC_high_ cell lines. Inhibition of TRIP13 largely impaired the proliferation of the TRIP13-high cell line H1437, while leaving the proliferation rates of TRIP13-low cell lines H1563 and H2122 unaffected. In addition, when TRIP13 is inhibited, H1437 shows the second largest reduction in the ability to form colonies. Together, these experiments lead us to conclude that TRIP13 expression alone in this cell line causes a phenotype that is similar to the GC_high_ cell lines. Interestingly, in contrast to DSB-marker γH2AX, the cell line H1437 (low GC gene score but high TRIP13) did not follow the RAD51 pattern (marking HR) of the GC_high_ cell lines, suggesting that the TRIP13 dependent increase in HR may interact with expression of other (germ line specific) genes. Nevertheless, we suggest that TRIP13 inhibition may be an effective strategy in the treatment of tumors that express TRIP13. This is supported by our result showing a decrease in RAD51 levels, but not the NHEJ proteins KU70 and Ligase IV, in all six LUAD cell lines upon treatment with DCZ0415. We found that the effect of TRIP13 inhibition during IR treatment may be independent from the initial level of *TRIP13* RNA expression, suggesting that combining TRIP13 inhibition with irradiation may be effective regardless of the initial TRIP13 expression levels.

GC genes have been proposed as ideal candidate targets in cancer treatment for two major reasons. First, because GC genes are responsible for germline-specific processes, solely targeting these processes should not harm other cells and thus lead to limited side-effects. Side effects may affect development, fertility, or none at all, depending on the expression profile of the target(s). Second, GC genes are hypothesized to contribute to the known hallmarks of cancer [26]. As a cancer is dependent on these features, targeting these oncogenic processes is more likely to effectively cripple a cancer, rather than inducing resistance through the selection of cancer cells following a treatment. TRIP13 appeared as a GC gene in our previous analyses due to high expression in primordial germ cells and throughout spermatogenesis [6, 7]. Its dual involvement in both the spindle assembly checkpoint and DNA repair makes it an appealing anticancer target. Despite a high RNA expression in the germline and many types of cancer, and low RNA expression in normal somatic tissues, TRIP13 is not strictly specific to cancer and the germline as it is also involved in the mitotic spindle assembly checkpoint. Therefore, it may not be an appropriate target in CAR T-cell therapies. However, inhibiting TRIP13 directly, especially when combined with radiotherapy or other DNA damaging agents such as cisplatin [37, 48, 49], could be a viable treatment modality to result in a higher mutational burden or lead to mitotic catastrophe. The clinical safety and efficacy of a TRIP13-targeted treatment remains to be investigated in future studies. Similar to the differential expression of TRIP13, there are many more (GC) genes that share a similar germline/cancer expression profile. Further investigation could focus on the role of GC genes in the human germline and soma, as targeting germline-specific processes such as meiosis in cancer treatment has the potential to severely limit side effects.

### Supplementary information

Supplementary data 1

Supplementary table and figures

Checklist

## Data Availability

All data generated or analyzed during this study are included in this published article [and its supplementary information files].
