# Supplementary material for: Germline specific genes increase DNA double-strand break repair and radioresistance in lung adenocarcinoma cells
Source: Cell Death Dis. 2024 Jan 12;15(1):38. doi: 10.1038/s41419-024-06433-y (PMC10786935; doi:10.1038/s41419-024-06433-y)

Liu et al 2023, Supplementary table 1 and figures S1-7

Supplementary Table 1: Primer sequences

Plasmid		
TRIP13 Double Nickase	CACGTGGAGGTGCATCAGCG	CGTTGGCGACTCGGCCACAC
TRIP13 CRISPR Activation	AACCTCGGCGCAGCGAGCGC	

Gene	Forward primer	Reverse primer	Product size (bp)
Gapdh	GTCTCCTCTGACTTCAACAGCG	ACCACCCTGTTGCTGTAGCCAA	130
Actb	ACCAGAGGCATACAGGGAC	CTAAGGCCAACCGTCAAAAG	100
Tuba1c	AGCGTGCCTTTGTTCACT	CTCATCCTCTCCGTCAGC	134
Trip13	CGGGTCCTGAGAAAACTCCC	CAAACTGCTTGTCCACTGCC	150
Sox2	GTTACGCGCACATGAACGG	GTAGGACATGCTGTAGGTGGG	206
Nanog	CCTGTGATTTGTGGGCCTGA	GGGTTGTTTGCCTTTGGGAC	156
Oct4	TGGGGGAAGAGTAGTCCTTTG	ATCTCCCCTTTCCATTCGGGA	179

Liu et al 2023, Supplementary table 1 and figures S1-7

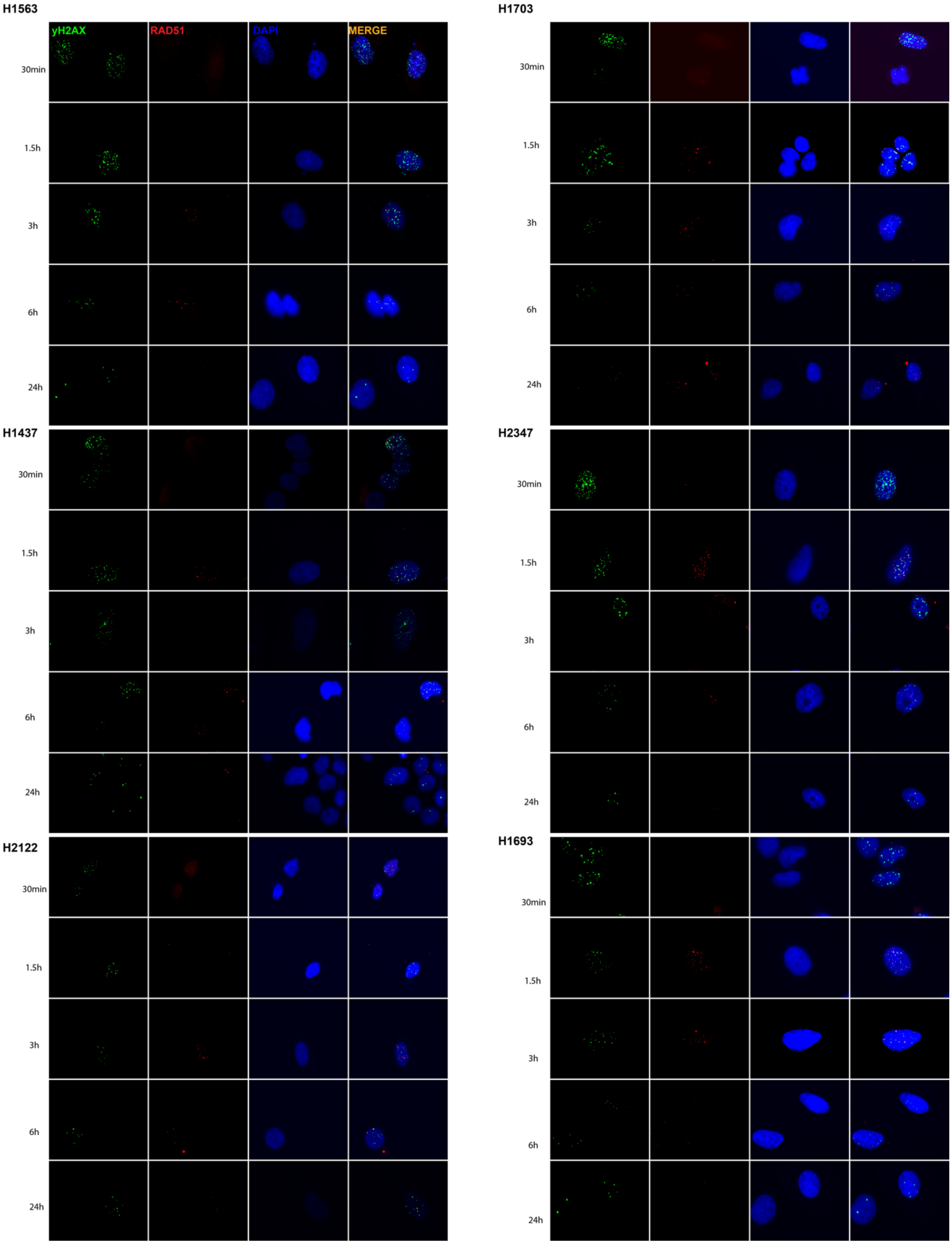

Figure S1. GC $_{high}$ cell lines repair double-stranded breaks more efficiently than GC $_{low}$ cell lines.

γ-H2AX (green); RAD51 (red) and DAPI (blue) staining of all six LUAD cell lines at several time points after 1 Gy of irradiation. Left panels: 3 GC $_{low}$ cell lines. Right panels: 3 GC $_{high}$ cell lines.

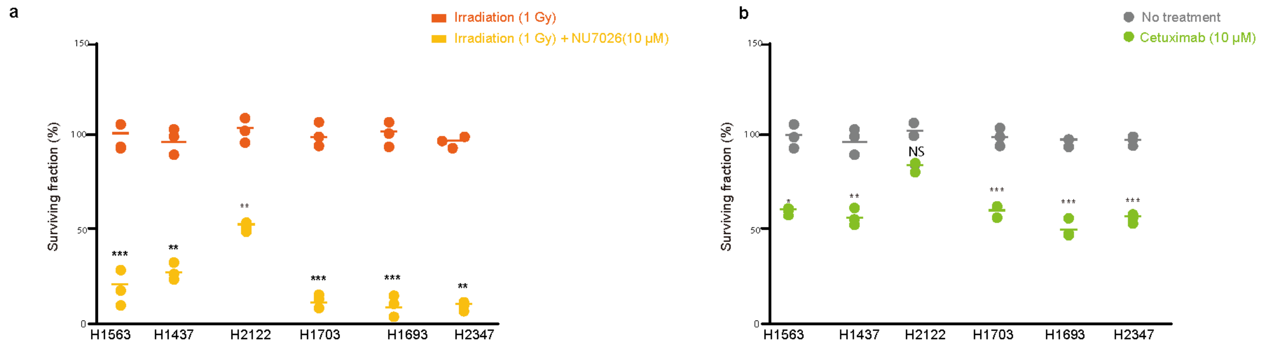

Figure S2. Relative survival of the LUAD cell lines in response to EGFR or DNA-PKcs inhibition.

a. *Relative survival of the LUAD cell lines 14 days after IR, with or without 10 μM NU7026 (DNA-PKcs inhibition).*

b. *Relative survival of the LUAD cell lines, with or without 10 days of treatment with 10 μM Centuximab. NS = not significant, * = p < 0,05. ** = p < 0,01. *** = p < 0,001.*

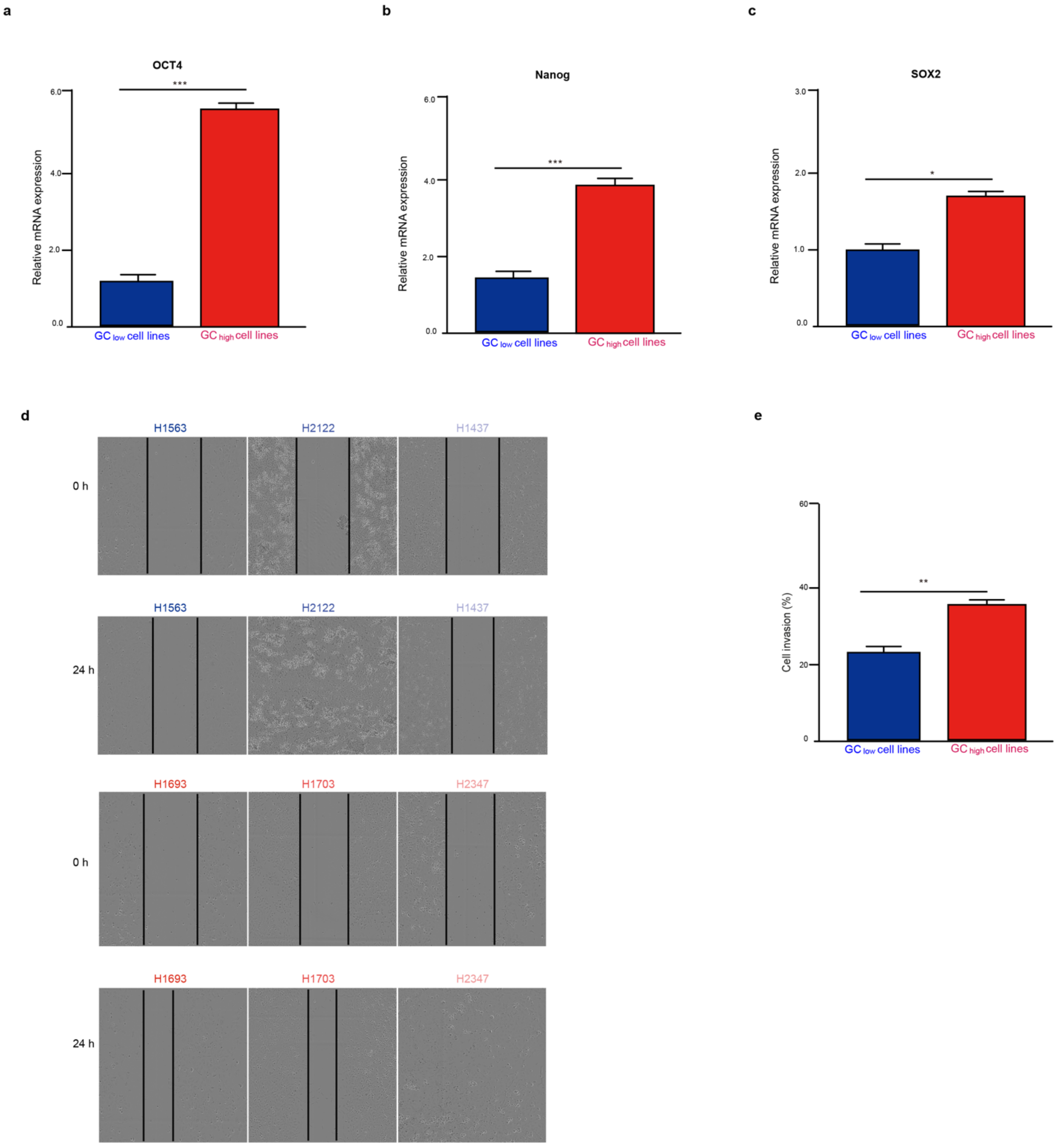

Figure S3. GC high cell lines show higher expression of pluripotency markers and higher invasion potential.

a, b, c. *OCT4, Nanog and SOX2 mRNA expression in GC $_{low}$ cell lines and GC $_{high}$ cell lines.*

d. *Representative light microscope images of cell scratch assay in six LUAD cell lines at 0h and 24h.*

e. *Cell invasion analysis in GC $_{low}$ cell lines and GC $_{high}$ cell lines using Image J（2.0）.*

*NS = not significant, * = p < 0,05 . ** = p < 0,01. *** = p < 0,001.*

Liu et al 2023, Supplementary table 1 and figures S1-7

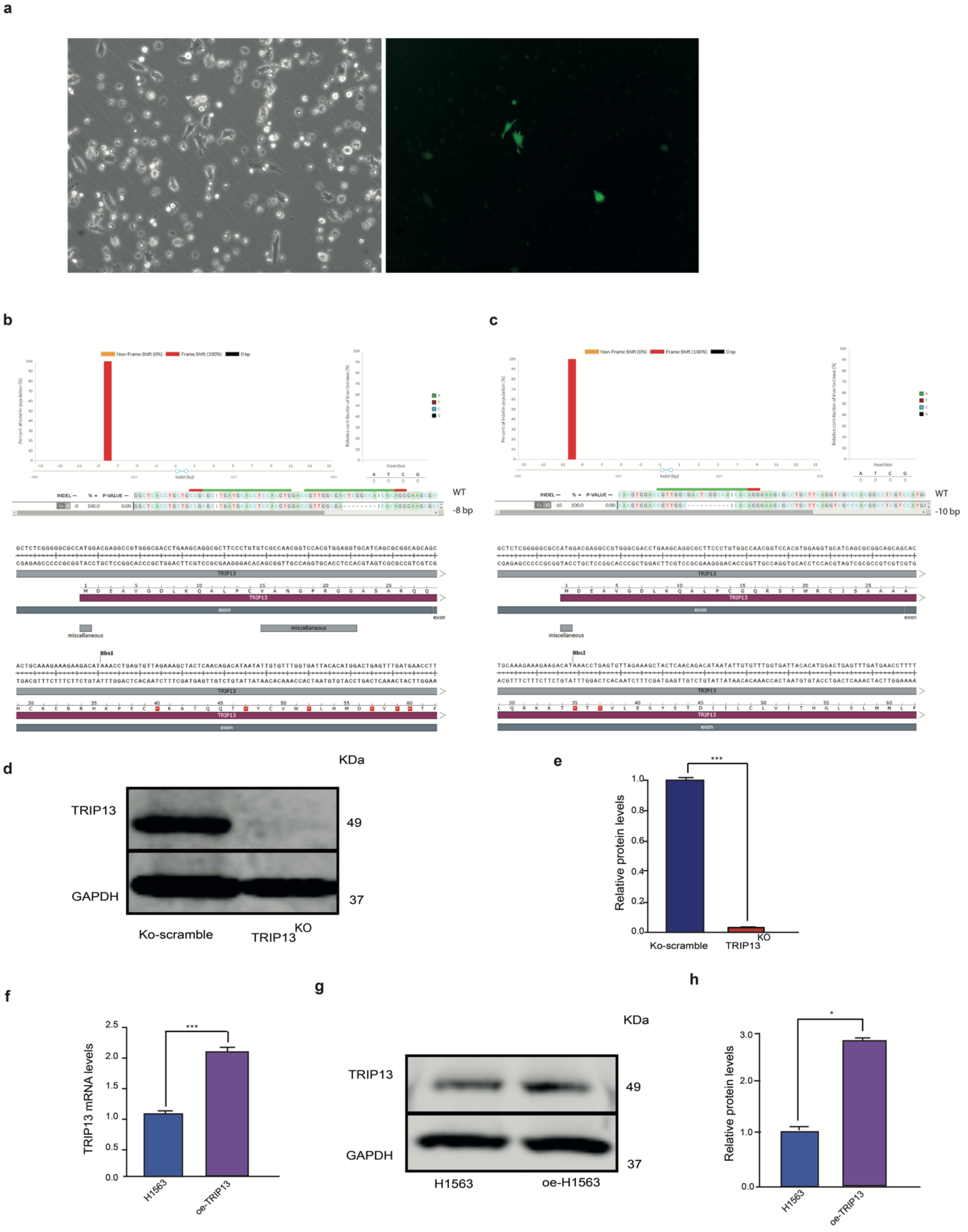

Figure S4. CRISPR-CAS9 *TRIP13* knockout and overexpression cell lines.

a. *H1703 cells 2 days after transfection with TRIP13 CRISPR-CAS9 plasmids.*

b. and c. *Sanger-sequencing analysis of the two TRIP13 alleles in the knockout cell line and location of the induced stop Codon.*

d*. Lysates from H1703 Ko-Scramble and* $TRIP13^{KO}$*, were immunoblotted using anti-TRIP13 and anti-GAPDH.*

e*. Band intensities were quantified and normalized to corresponding GAPDH.*

f*. TRIP13 mRNA expression in H1563 and oe-TRIP13.*

g*. Lysates from H1563 and oe-TRIP13 were immunoblotted using anti-TRIP13 and anti-GAPDH.*

h*. Band intensities were quantified and normalized to corresponding GAPDH. * = p < 0,05. ** = p < 0,01. *** = p < 0,001*

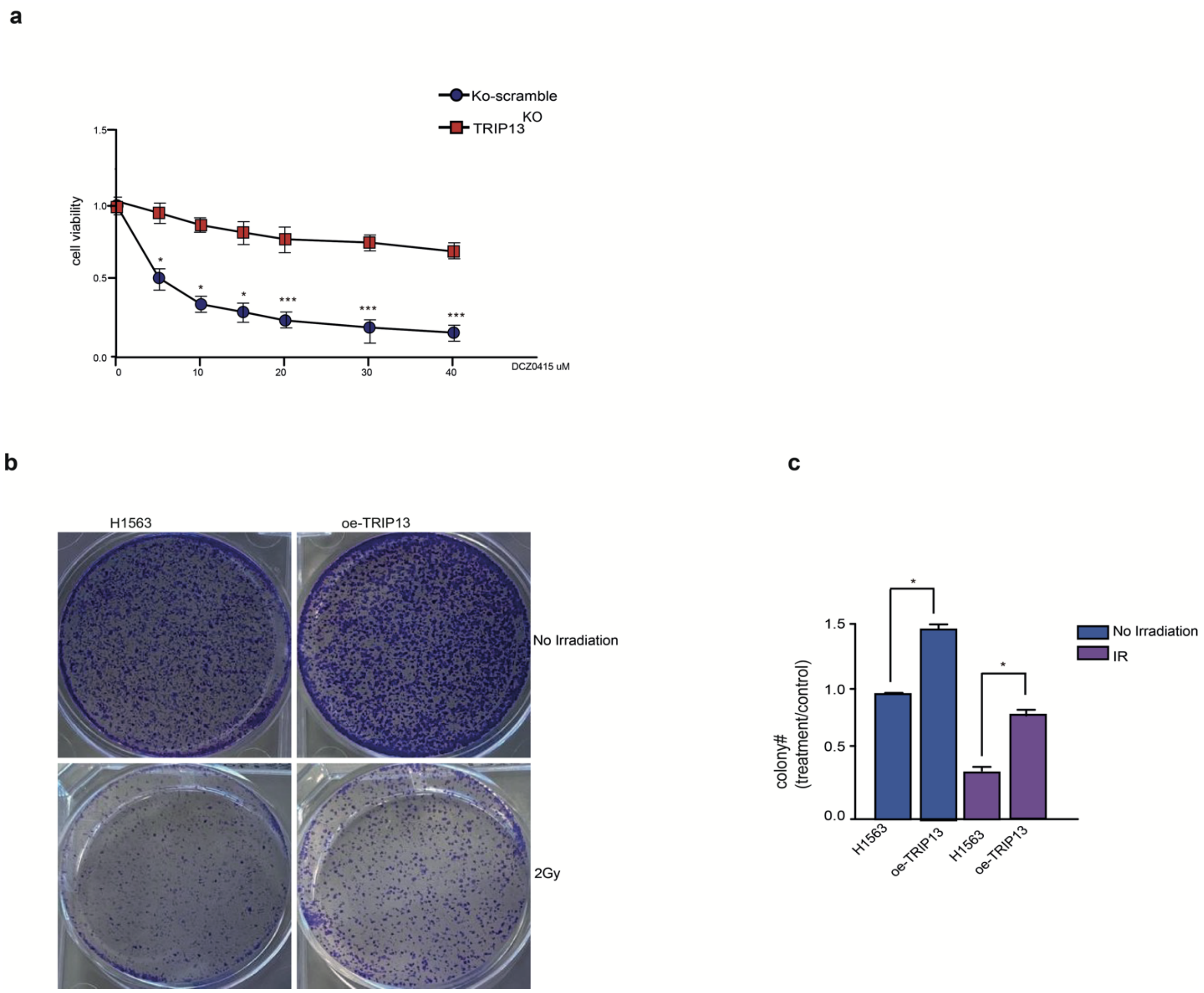

Figure S5. Cell survival after treatment with DCZ0415 and irradiation.

a. *Cell viability assay for H1703 Ko-Scramble and ($TRIP13^{KO}$) at different concentrations of DCZ0415.*

b. *Overexpression of TRIP13 significantly increases radioresistance of H1563 cells; example, surviving colonies at 14 days after 2Gy or no irradiation.*

c. *The percentage of proliferative cells or colony formation normalized to that of the control group. * = p < 0,05. ** = p < 0,01. *** = p < 0,001.*

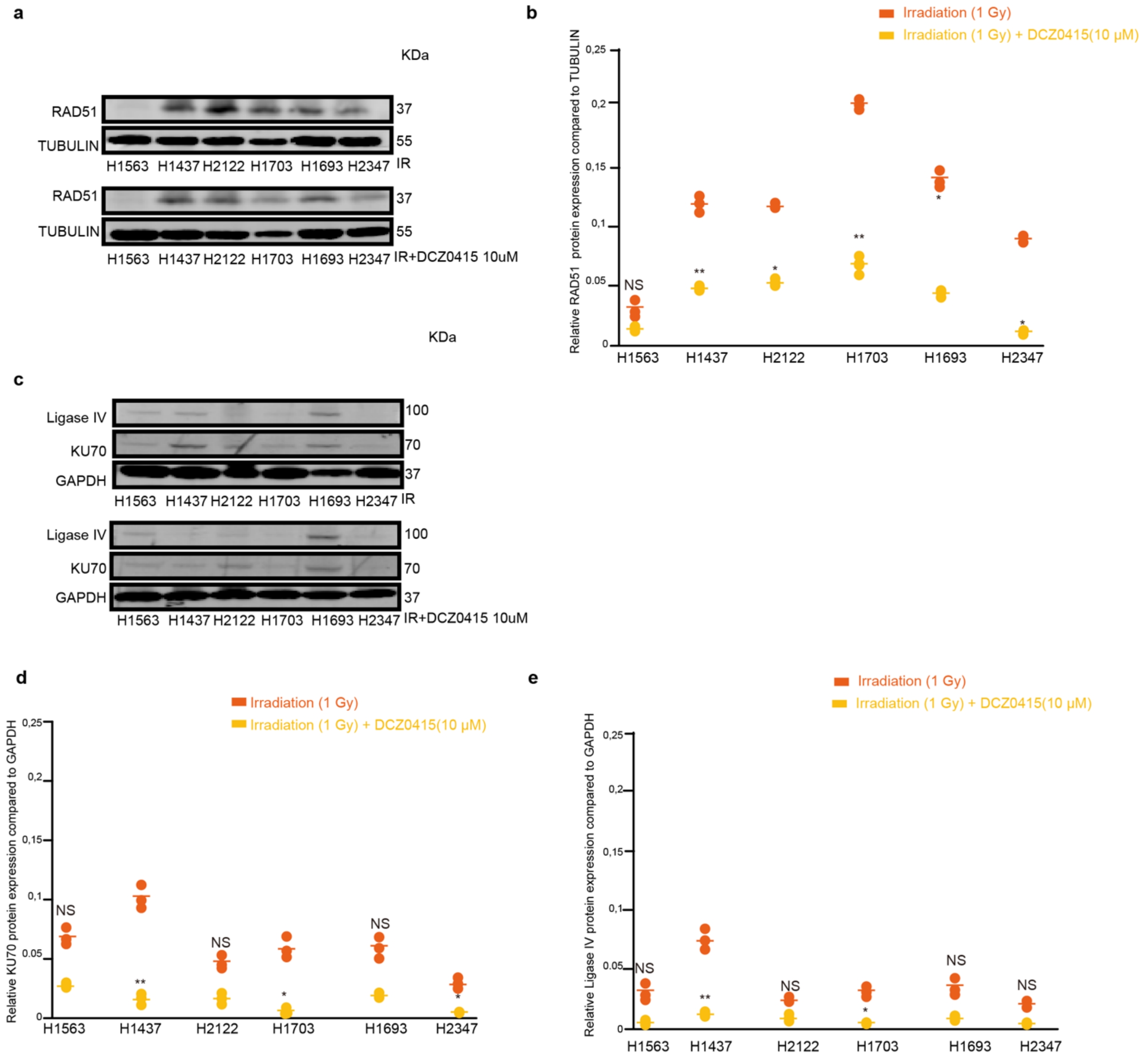

Figure S6. TRIP13 inhibition decreases RAD51 induction after irradiation.

a. *Western blot showing RAD51 levels in the six cell lines after 1 Gy IR, with or without 10 μM DCZ0415, normalized against TUBULIN.* ***b.*** *Quantified RAD51 protein levels relative to TUBULIN* ***c****. Western blot showing KU70, Ligase IV and GAPDH protein levels in the six cell lines after 1 Gy IR, with or without 10 μM DCZ0415.* ***d****. Quantified KU70 protein levels relative to GAPDH. after 1 Gy IR, with or without 10 μM DCZ0415.* ***e.*** *Quantified Ligase IV protein levels relative to GAPDH, after 1 Gy IR, with or without 10 μM DCZ0415. NS = not significant, * = p < 0,05. ** = p < 0,01. *** = p < 0,001.*

Liu et al 2023, Supplementary table 1 and figures S1-7

Figure S7. Original blots

Figure 5c

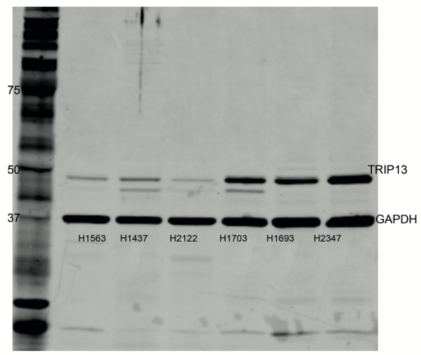

Figure S4a

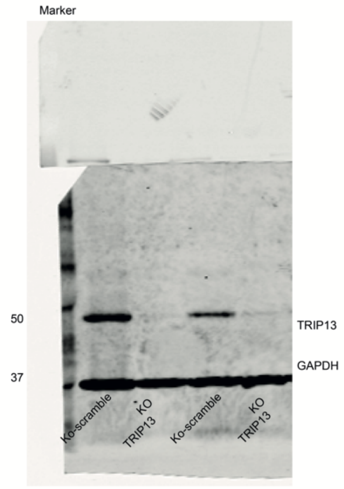

Figure S4g

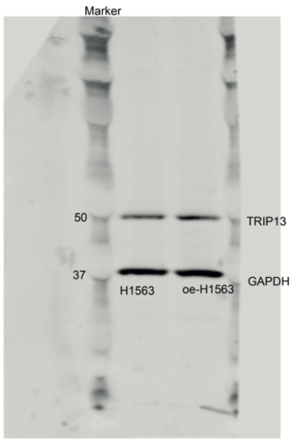

Figure S6a

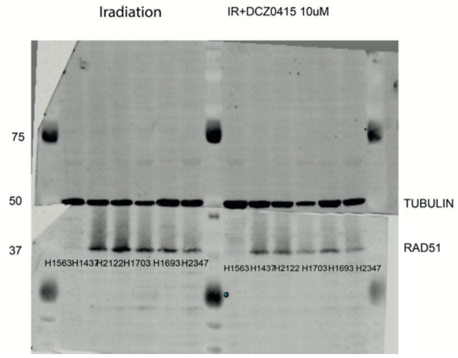

Figure S6c

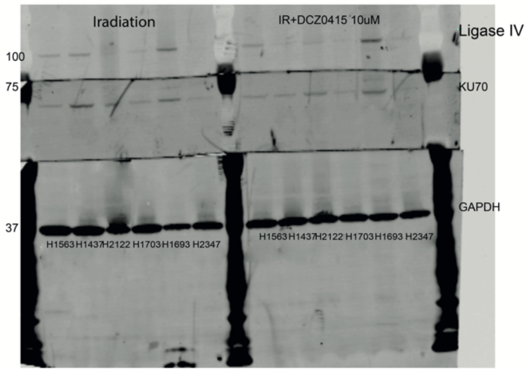

Supplement: Supplementary file 2 — Supplementary table and figures [file 41419_2024_6433_MOESM2_ESM.pdf]
